# A Neglected Parasite: *Macracanthorhynchus hirudinaceus*, First Report in Feral Pigs in a Natural Park of Sicily (Southern Italy)

**DOI:** 10.3389/fvets.2021.659306

**Published:** 2021-04-28

**Authors:** Sergio Migliore, Roberto Puleio, Gabriella Gaglio, Domenico Vicari, Salvatore Seminara, Elio Rodolfo Sicilia, Paola Galluzzo, Valentina Cumbo, Guido Ruggero Loria

**Affiliations:** ^1^Istituto Zooprofilattico Sperimentale della Sicilia “A. Mirri”, Palermo, Italy; ^2^Dipartimento di Scienze Veterinarie, Università degli Studi di Messina, Messina, Italy; ^3^Centro Interuniversitario di Ricerca in Parassitologia (CIRPAR), Naples, Italy; ^4^Azienda Sanitaria Provinciale di Palermo, Palermo, Italy

**Keywords:** wildilife, parasites, *Macracanthorhynchus hirudinaceus*, feral pigs, zoonoses

## Abstract

Sanitary management and population control of feral pigs remains a major problem in public health, particularly in natural parks where hunting is prohibited and the extensive farming of livestock is common. *Macracanthorhynchus hirudinaceus* is a zoonotic parasite species with a worldwide distribution of which the natural definitive hosts are primarily pigs and wild boars (*Sus scrofa*). The present study describes the main anatomo-pathological and parasitological findings in the first case of *M. hirudinaceus* in feral pigs in the Madonie park in Sicily (Southern Italy). Overall, 52 acanthocephalans were collected from the small intestine of four infected feral pigs. The prevalence among the 36 examined animals was 11.1% with a mean Abundance (mA) and mean Intensity (mI) of 1.4 and 13, respectively. Pathological examination revealed grossly visible nodules on the external surface of the intestines, corresponding to the proboscis of *M. hirudinaceus* attached deeply into the intestinal wall. In these sites, severe inflammatory reactions in the tissue involved and the destruction of normal intestinal architecture, as well as necrosis and ulceration in the mucosa, submucosa, and part of the *muscolaris mucosae* were described. This is the first official report of this neglected zoonosis in Italy, in particular in a natural park where the extensive farming of domestic pigs is practiced. This could favor the spread of this parasite in domestic animals and the environment, increasing the accidental risk of infection in human residents of these areas.

## Introduction

Feral pigs (*Sus scrofa*) are animals widespread in almost all countries of the world that generally live in woodlands and parks or, more often, are kept in extensively managed farms. Feral pigs have a recognized severe impact due to their extraordinary reproductive rate and their impact on local biodiversity and soil often damaged by large groups of these ungulates (1). In addition, this species today represents an uncontrolled reservoir of different infectious diseases either for other ungulates or for humans (zoonosis) and domestic animals (2). Pigs are omnivorous, and their diet also contains all animal proteins that they can easily catch, including arthropods, birds, mice, and small amphibious reptiles, and for this reason they can host a large number of parasitic infections. *Macracanthorhynchus hirudinaceus*, with a length of 7–14 cm and a width of 3.0–3.7 cm in males and surprisingly from 11 up to 40 cm and 3.0–5 cm in females, is considered the largest acanthocephalan species (3). The natural definitive hosts are primarily domestic (or feral) pigs and wild boars worldwide in which Acanthocephalans harboring in its intestinal tract (4). When the female worms reach maturity, they typically excrete eggs containing larval acanthors in feces. The beetle, usually the dung beetle (*Geotrupes stercorarius*), serves as the intermediate host (5) that ingests the eggs in which the acanthor develops into an acanthella and then a cystacanth. The definitive host is infected orally by uptake of infected beetles or parts of them containing an infectious cystacanth, which will develop into adult male and female Acanthocephalans (4). Carnivores and primates, including humans, may represent accidental definitive hosts (6, 7).

The prepatent period in pigs is 8–12 weeks, followed by an asymptomatic, subclinical course or aspecific symptoms such as enteritis, peritonitis, diarrhea, malnutrition, and abdominal pain (4). *Macracanthorhynchus hirudinaceus* infections in wild boars are reported in Turkey (8), Iran (3, 9, 10), and Spain (11–13); in the latter it is considered in increasing trend (14).

Sanitary management of feral pigs in Sicily (Southern Italy) remains a major problem, particularly in natural areas or parks where extensive farming allows uncontrolled contacts with wild species (15). Feral pig populations in Sicily have increased dramatically over past decades, and they can serve as reservoirs for a number of bacteria, viruses, and parasites transmissible to humans and domestic animals through direct interaction or indirectly through contamination of the environment. In this paper, we report the first case of *M. hirudinaceus* infestation in a Sicilian feral pig population and the main pathological and parasitological findings in the infected animals.

## Methods and Materials

### Study Area

This study was carried out in the areas of the Madonie Natural Park in Sicily, between Palermo and Cefalù that covers 161.76 square kilometers, which lies on the geographical coordinates of 37° 53′N 14° 01′E. The park includes the Madonie Mountains and urbanized areas constituted by dozens of little villages and small towns. One of the park's most notable natural features is the beech trees forest found from altitude of 1,500 m up to the tops. These are the most southern beech forests in Europe (16). This environmental feature makes this area a suitable habitat for feral pigs and other wild animals. In this context, extensive farming constituted by mixed herds of ruminants together with swine, grazing in common areas still represent a sustainable (and ecological) opportunity for local economy.

### Animals

Laboratory investigations were performed from 2017 to 2018 on 36 carcasses of feral pigs trapped during the development of activities related to the feral pig population control plan in the protected area of Madonie Park. The age was established based on the development of teeth (17). We evaluated the eruption of the three main molars (M1, M2, and M3) that erupt in a known sequence: M1 erupts from 4 to 6 months, M2 erupts at 12 months, the first cusp of M3 at 24 months, and the third cusp at 42 months. Under this scheme, 23 animals were <1 year of age, and 13 were in a range of 1–3 years. Animals were identified as 21 females and 15 males, while their body weight ranged from 12 to 67 kg. The animals were culled following Council Regulation (EC) No 1099/2009 of September 24, 2009, on the protection of animals at the time of killing.

### Parasitological Sampling

At necropsy, the gut was longitudinally opened and examined for parasitological investigations and related lesions. Parasites were isolated using total worm count technique (18). All helminths were put in a 70% alcohol solution with 5% added glycerin. Prevalence values were calculated at a 95% confidence interval (CI).

Identification was performed using classical morphological keys (19, 20). Some specimens of *M. hirudinaceus* were processed for Scanning Electron Microscopy observation (SEM). These were dehydrated through a graded series of ethanol from 70 to 100%, dried with liquid CO_2_ according to the critical point method and mounted onto stubs. Mounted specimens were then sputter-coated with a palladium gold layer (20 nm ± 5%) and observed with an SEM Zeiss EVO 10 MA (Carl Zeiss MicroscopyGmbH, Jena, Germany). Basic epidemiological data were calculated according to Bush et al. (21).

### Tissue Sampling and Examination

Portions of tissues from the gastrointestinal tract, including some sections (0.5 × 2 × 4 cm) taken from the areas where the nodular lesions were observed, were fixed in 10% buffered formalin. Serial sections of paraffin embedded tissues of 5–6 μm thickness were cut using a microtome, stained with hematoxylin-eosin (HE), and studied under light microscope.

## Results

### Parasitological Examination

A coinfection of two species of parasites was found in four animals that showed typical nodular lesions in the intestinal tract. Parasites were identified as *M. hirudinaceus* (52 worms) and *Ascaris suum* (16 worms). The overall prevalence of *M. hirudinaceus* and *A. suum* in the sample was 11.1% (4 pigs out of 36) (95% CI: 0.01–0.21) and 16.6% (6 pigs out of 36) (95% CI: 0.047–0.287), respectively. The mean Abundance (mA) and the mean Intensity (mI) for *M. hirudinaceus* were 1.4 and 13%, while for *A. suum* the figures were 0.4 and 2.7%. Among the 52 specimen of *M. hirudinaceus* collected, males constituted 44% (23 out of 52) and females 56% (29 out of 52) of the sample. Total body length ranged from 4.9 to 9.1 cm and 6.1 to 37.4 cm in male and female worms, respectively. Young acanthocephalans (measured 110–130 mm) were observed in few cases. The highest intensity of infestation of *M. hirudinaceus* per sample was 19 worms. Subjects that did not show nodular lesions showed a singular parasitic infection with 23 and 18 individuals, respectively of *A. suum*.

**Figure 1:**
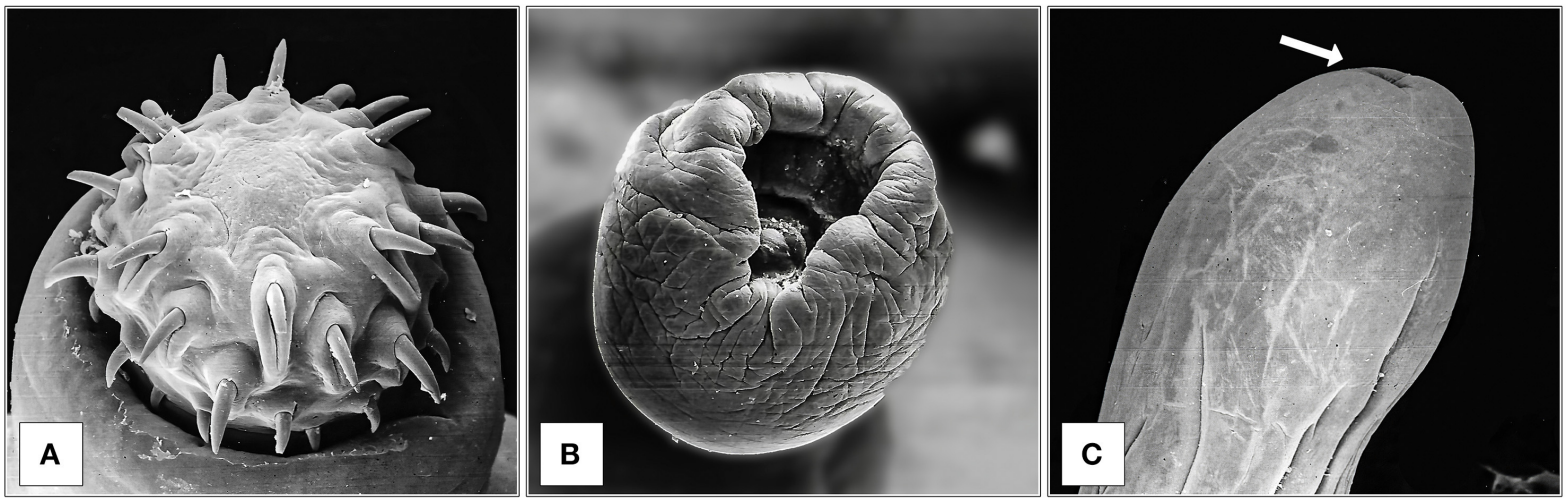
SEM observation of *M. hirudinaceus*: Cephalic region **(A)**; Copulatory bursa of male **(B)**; Caudal region of female **(C)**, and genital pore (arrow).

SEM observation of *M. hirudinaceus* showed detailed surface morphology. The cranial region was characterized by the retractile proboscis with typical hooks and epidermal elevation at their insertion point (Figure 1A). The caudal end of males showed well-developed copulatory bursa (Figure 1B). The caudal region of female specimens had a rounded end and a visible genital pore (Figure 1C).

### Anatomo-Pathological Findings

**Figure 2:**
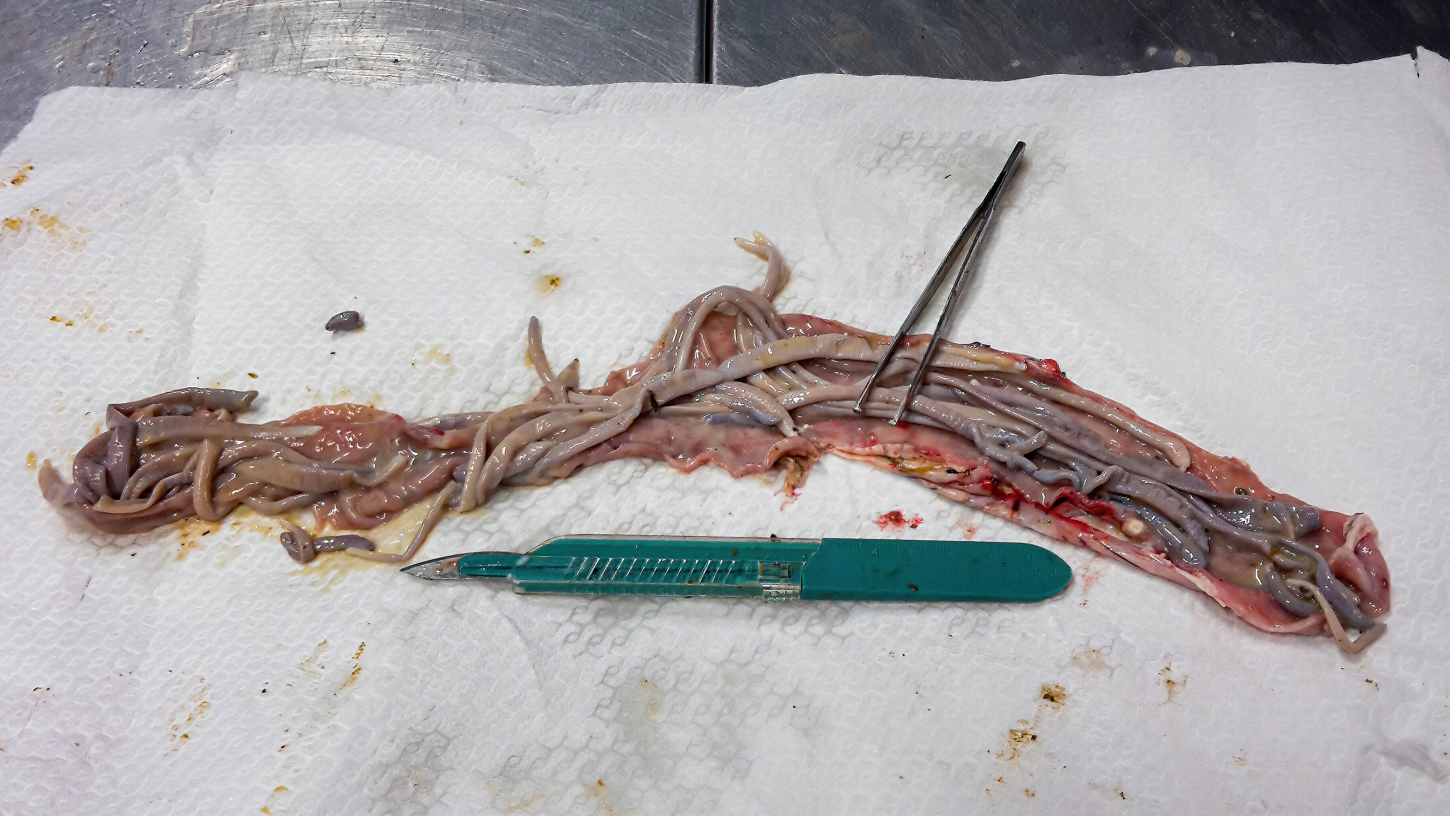
Massive infestation of helminths (*M. hirudinaceus* and *A. suum*) in a young female feral pig.

**Figure 3:**
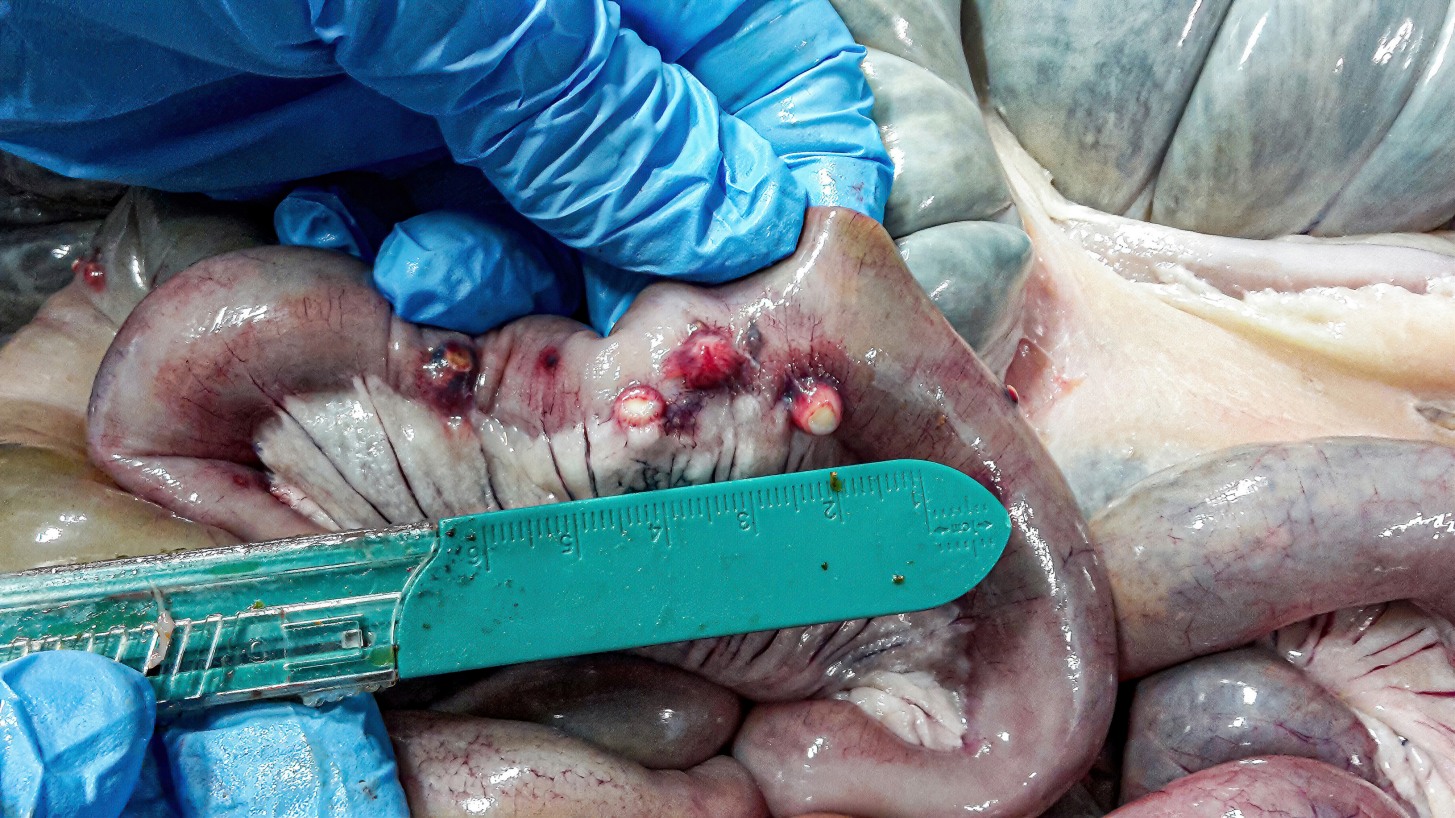
Nodules (~5 mm) on the external surface of the small intestine surrounded by a hyperemic-hemorrhagic halo.

**Figure 4:**
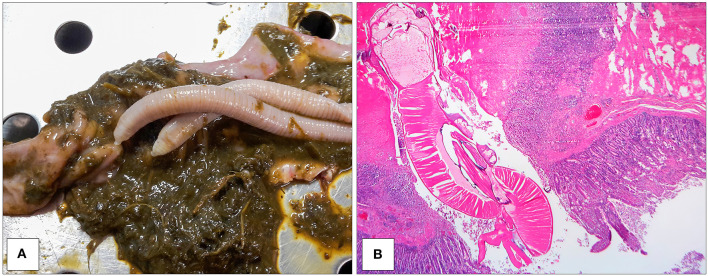
Specimens of *M. hirudinaceus* attached in the intestinal mucosa **(A)**. Intestine showing dense compressed necrotic tissue adjacent to the apical portion of helminth. The tissue surrounding this area contained numerous lymphocytes and monocytes. Eroded villi and necrotic epithelial cells around the trunk of the worm (EE 2.5X) **(B)**.

All culled pigs showed a good state of nutrition and a normal presence of subcutaneous fat, none of them showed pathognomonic lesions due to bacterial or viral infections. However, a group of six animals (6 out of 36) captured on the same site (Volpignano Hill), aged <1 year old and a weight ranging from 23 to 35 kg, showed dilatation of the intestinal lumen and obstruction due to a massive presence of parasites. For these subjects, a particular physical examination of the gastrointestinal tract was performed. In the intestinal lumens, a significant infestation of helminths (up to 19 specimens for each animal) with approximate sizes ranging from 4 to 40 cm in length were detected (Figure 2). Four pigs (1 male and 3 females) showed similar pathological findings: mesenteric lymph nodes were enlarged, and the serosa of the small intestine showed a variable number of roundish small nodules (~5 mm) surrounded by a hyperemic-hemorrhagic halo (Figure 3). In the mucosal surface of the small intestine, the nodules showed a slight relief, centrally umbilicated, where, in most of these, the specimen of helminth was attached by means of its apical portion entirely embedded in the nodule (Figure 4A).

Two other pigs (males) did not show external lesions in the gastrointestinal mucosa but revealed a significant presence of helminths.

The histopathology of the nodules in the small intestine showed severe damage of the tissue with an intense inflammatory reaction. At the site of attachment, the normal architecture of the intestine was disrupted, and the worm had penetrated the mucosa, submucosa, and part of the *muscolaris mucosae* (Figure 4B). Intestinal villi and their epithelium were destroyed at that site. The underlying mucosa and submucosa were also severely damaged. In addition, partial perforation was observed in the place of the parasite attachment that involved all layers of the intestinal wall apart from the serosa. A layer of fibro-necrotic debris surrounded the apex of worm, where it was embedded in the *muscularis* (Figure 4B). The homogeneous, amorphous layer stained bright pink with hematoxylin and eosin, indicating the presence of fibrin at the site of severe connective tissue injury. Monocytes and lymphocytes debrided and disrupted muscle tissue and lined the internal edge of the fibrin layer. The remaining tissue in the nodule showed increased interstitial space and muscle fibers running irregularly. Evidence of the chronicity of the lesion included the preponderance of monocytes, lymphocytes, macrophages, fibrosis, and neovascularization (Figure 4B).

## Discussion

The presence of *M. hirudinaceus* in feral pigs caught or hunted in the wild is not a surprising finding; however, this is the first official report of this neglected zoonosis in Italy. The prevalence recorded in our study (11.1% of the culled pigs in 2017–2018) is significantly considered within the massive increase of the feral pig population in Italy and Sicily. In this study, we reported *M. hirudinaceus* only in young feral pigs (<1-yr-old), and similar results were reported in Spain (13). However, in Mowlavi et al. (3) study, adult wild boars were more infected than young animals. This dissimilarity could be explained by the low sample size of our study.

Pathological studies showed severe damage of the tissue with an intense inflammatory reaction in deep small intestine wall. At the site of attachment, the parasite's hooks penetrated into the submucosal layer of intestine and caused severe damage of villi and their epithelium. The pathological findings of this study are in line with other studies (3, 22). From the inspection point of view, the macroscopic lesions observed should be considered in a differential diagnosis of intestinal tuberculosis, often reported in feral pigs, especially in regions where tuberculosis is endemic.

Different studies have reported *M. hirudinaceus* infection rates in wild boar: 18.5% in the Turkish province of Bursa (8), 41.6% in Luristan (9), 64% in Khuzestan (3), and 52% in Busherhr (10). Furthermore, in previous Spanish studies, prevalence was 33.3% in southern Spain (11), 61.2% in central Spain (12), and around 21% in the Valencian Community (13, 14).

Despite these prevalence rates, no human cases in these countries were reports. Human infections were observed in China (23, 24); Thailand (25–30), and Russia (31).

*Macracanthorhynchus hirudinaceus* eggs are very resistant in the environment and remain infectious for up to 3 years, tolerating both drought and below-zero temperatures (32). Currently, the reduced number of reports of human cases is probably a consequence of the low endemicity of the parasite due to the changes in pig farming and improved health education (33).

The date of wild boar extinction in Sicily is not certain, but it may have occurred toward the end of the 19th century due to unrestricted hunting. The last written reports of the wild boar presence in Sicily, as an endemic species, date back to the end of the 19th century (34, 35) and more precisely to 1868 in the Madonie mountains (36). However, in the 70s, small groups of wild boar of unclear genetic origin were introduced into public or private farms belonging Madonie and Nebrodi Mountains. In the following years, some of these animals escaped from captivity with constant and progressive colonization of new areas, also due to the absence of natural predators and hunting activity (because of the Parks). The crossbreeding between wild subspecies and local domestic pigs belonging to those areas has created the current feral pig population, characterized by an absence of any ecological link with the environment and a much higher natality rate than wild boars. The last official census, dating back to 2012, reports a presumable size of the population of the Madonie Mountains around 3,000–5,000 heads, but today the size of feral pig population in Sicily and its sanitary status remain unknown.

Diseases that arise from livestock-wildlife interaction are of paramount importance and must be an area of focus for public and veterinary health systems (37). Feral pigs have become of increasing concern as a potential veterinary and public health threat for cross-species transmission (38). In some parts of the world, feral pigs have been identified as an important reservoir for epidemic diseases to other domestic animals with serious socio-economic consequences (39). Feral pigs have been also implicated in the transmission of zoonotic diseases, such as hepatitis E virus (HEV) (40), trichinellosis (41), swine influenza virus (42), and Japanese encephalitis virus (43). In addition, feral pigs have been identified as a contributor to O157:H7 *Escherichia coli* contamination in watersheds (44). Interest in the role that feral pigs may play in foodborne illness has also increased after recent outbreaks of *Salmonella* spp. in spinach and other leafy greens that were traced back to farms in areas with feral pig populations (45, 46).

In this paper, we report for the first time *M. hirudinaceus* infection in a feral pig population in a natural park where the extensive farming of domestic pigs is practiced. The presence of neglected and zoonotic parasites such as *M. hirudinaceus* in feral pigs and therefore in the environment points to the need of efficient sanitary controls in these population. Extensive farming allows, in fact, uncontrolled contacts with feral pigs, which are generally managed without anthelmintics, food supplementation, and routine veterinary monitoring. This could favor the spread of this neglected parasitosis in domestic animals and the environment, increasing the accidental risk of infection in human residents of these areas.

## Data Availability Statement

The original contributions presented in the study are included in the article/supplementary material, further inquiries can be directed to the corresponding author/s.

## Ethics Statement

Ethical review and approval was not required for the animal study because Feral pigs analyzed in this study were culled according to Council Regulation (EC) No 1099/2009 of 24 September 2009 on the protection of animals at the time of killing.

## Author Contributions

SM and GL: conceptualization. RP and GG: methodology. SM, RP, and GG: formal analysis. RP, GG, ES, and DV: investigation. PG and VC: data curation. SM: writing—original draft preparation. SM, RP, GG, and GL: writing-review and editing. SS and GL: supervision. All authors have read and agreed to the published version of the manuscript.

## Conflict of Interest

The authors declare that the research was conducted in the absence of any commercial or financial relationships that could be construed as a potential conflict of interest.
